# Tailored HIV programmes and universal health coverage

**DOI:** 10.2471/BLT.18.223495

**Published:** 2019-09-27

**Authors:** Charles B Holmes, Miriam Rabkin, Nathan Ford, Peter Preko, Sydney Rosen, Tom Ellman, Peter Ehrenkranz

**Affiliations:** a Georgetown University School of Medicine, 3900 Reservoir Rd NW, Washington, DC 20007, United States of America (USA).; b ICAP at Columbia University, Mailman School of Public Health, New York, USA.; c Department of HIV and Global Hepatitis Programme, World Health Organization, Geneva, Switzerland.; d Health Economics and Epidemiology Research Office, University of the Witwatersrand, Johannesburg, South Africa.; e Medical Department, Médecins Sans Frontières, Cape Town, South Africa.; f TB and HIV, Bill & Melinda Gates Foundation, Seattle, USA.

## Abstract

Improvements in geospatial health data and tailored human immunodeficiency virus (HIV) testing, prevention and treatment have led to greater microtargeting of the HIV response, based on location, risk, clinical status and disease burden. These approaches show promise for achieving control of the HIV epidemic. At the same time, United Nations Member States have committed to achieving broader health and development goals by 2030, including universal health coverage (UHC). HIV epidemic control will facilitate UHC by averting the need to commit ever-increasing resources to HIV services. Yet an overly targeted HIV response could also distort health systems, impede integration and potentially threaten broader health goals. We discuss current approaches to achieving both UHC and HIV epidemic control, noting potential areas of friction between disease-specific microtargeting and integrated health systems, and highlighting opportunities for convergence that could enhance both initiatives. Examples of these programmatic elements that could be better aligned include: improved information systems with unique identifiers to track and monitor individuals across health services and the life course; strengthened subnational data use; more accountable supply chains that supply a broad range of services; and strengthened community-based services and workforces. We argue that the response both to HIV and to broader health threats should use these areas of convergence to increase health systems efficiency and mitigate the harm of any potential decrease in health funding. Further investments in implementation and monitoring of these programme elements will be needed to make progress towards both UHC and HIV epidemic control.

## Résumé

Les améliorations des données sanitaires géospatiales et la personnalisation du dépistage, de la prévention et du traitement du virus de l'immunodéficience humaine (VIH) ont permis de développer le micro-ciblage de la réponse au VIH, en fonction du lieu, du risque, de la situation clinique et de la charge de morbidité. Ces approches sont prometteuses pour lutter contre l'épidémie de VIH. Dans le même temps, les États membres des Nations Unies se sont engagés à atteindre des objectifs plus larges de santé et de développement d'ici 2030, notamment la couverture sanitaire universelle. Cette dernière sera facilitée par la lutte contre l'épidémie de VIH, qui réduira la nécessité de consacrer toujours plus de ressources aux services liés au VIH. Cependant, une réponse au VIH trop ciblée pourrait également distordre les systèmes de santé, empêcher leur intégration et potentiellement nuire aux objectifs de santé plus vastes. Nous abordons ici les approches actuelles en matière de couverture sanitaire universelle et de lutte contre l'épidémie de VIH, en notant les points de friction potentiels entre un micro-ciblage spécifique à certaines maladies et des systèmes de santé intégrés, ainsi que les opportunités de convergence qui pourraient être bénéfiques aux deux initiatives. Parmi les éléments de programmes qui pourraient être mieux coordonnés, nous pouvons citer: l'amélioration des systèmes d'information avec des identifiants uniques permettant de suivre les personnes dans leur parcours de soins et tout au long de leur vie; la plus grande utilisation des données infranationales; la responsabilisation des chaînes d'approvisionnement qui fournissent un grand nombre de services; et le renforcement des services et des intervenants communautaires. Nous soutenons que la réponse au VIH et à d'autres menaces sanitaires devrait exploiter ces domaines de convergence pour accroître l'efficacité des systèmes de santé et atténuer le préjudice d'une éventuelle baisse des fonds alloués à la santé. Il sera nécessaire d'investir davantage dans la mise en œuvre et le suivi de ces éléments de programmes pour avancer, aussi bien vers la couverture sanitaire universelle que dans la lutte contre l'épidémie de VIH.

## Resumen

Las mejoras en los datos geoespaciales de salud y las pruebas, la prevención y el tratamiento adaptados al virus de la inmunodeficiencia humana (VIH) han conducido a una mayor focalización de la respuesta al VIH, basada en la ubicación, el riesgo, el estado clínico y la carga de la enfermedad. Estos enfoques son prometedores para lograr el control de la epidemia del VIH. Al mismo tiempo, los Estados Miembros de las Naciones Unidas se han comprometido a alcanzar objetivos de salud y desarrollo de mayor alcance para 2030, incluida la cobertura universal de salud (universal health coverage, UHC). El control de la epidemia del VIH facilitará la UHC porque evitará la necesidad de comprometer recursos cada vez mayores para los servicios del VIH. Sin embargo, una respuesta al VIH demasiado específica también podría distorsionar los sistemas de salud, impedir la integración y amenazar potencialmente los objetivos de salud de mayor alcance. Se discuten los enfoques actuales para lograr tanto la atención primaria de salud como el control de la epidemia del VIH, se señalan las posibles áreas de fricción entre la focalización específica de la enfermedad y los sistemas integrados de salud, y se destacan las oportunidades de convergencia que podrían mejorar ambas iniciativas. Entre los ejemplos de estos elementos programáticos que podrían alinearse mejor se incluyen: sistemas de información mejorados con identificadores únicos para hacer un seguimiento y monitoreo de las personas a través de los servicios de salud y el curso de la vida; el fortalecimiento del uso de datos a nivel subnacional; cadenas de suministro más responsables que proveen una amplia gama de servicios; y el fortalecimiento de los servicios en la comunidad y las fuerzas de trabajo. Se argumenta que la respuesta tanto al VIH como a las amenazas para la salud en general debe utilizar estas áreas de convergencia para aumentar la eficiencia de los sistemas de salud y mitigar el daño de cualquier posible disminución en la financiación de la salud. Se necesitarán más inversiones en la ejecución y el monitoreo de estos elementos del programa para avanzar tanto en el control de la epidemia de VIH como en la UHC

## ملخص

أدت التحسينات في بيانات الصحة الجغرافية المكانية، واختبار فيروس نقص المناعة البشرية (HIV)، والوقاية منه وعلاجه، إلى زيادة الاستهداف الدقيق للاستجابة لفيروس نقص المناعة البشرية، بناءً على الموقع والمخاطر والحالة السريرية وعبء المرض.تمثل هذه الأساليب وعداً بتحقيق السيطرة على وباء فيروس نقص المناعة البشرية.وفي نفس الوقت، التزمت الدول الأعضاء في الأمم المتحدة بتحقيق أهدافاً أوسع للصحة وللتنمية بحلول عام 2030، بما يشمل التغطية الصحية الشاملة (UHC).سوف تؤدي مكافحة وباء فيروس نقص المناعة البشرية (HIV) إلى تسهيل التغطية الصحية الشاملة عن طريق تجنب الحاجة إلى تخصيص موارد متزايدة دائماً لخدمات فيروس نقص المناعة البشرية.إلا أن الاستجابة المفرطة المستهدفة لفيروس نقص المناعة البشرية قد تؤدي أيضاً لتشويه الأنظمة الصحية، وتعويق التكامل، ومن المحتمل أن تهدد الأهداف الصحية الأوسع نطاقاً.نحن نناقش الأساليب الحالية لتحقيق كل من التغطية الصحية الشاملة، ومكافحة وباء فيروس نقص المناعة البشرية، مع ملاحظة مجالات التداخل المحتملة بين الاستهداف الدقيق للأمراض المحددة، والنظم الصحية المتكاملة، وتسليط الضوء على فرص التقارب التي يمكن أن تعزز كلتا المبادرتين.من أمثلة هذه العناصر البرمجية التي يمكن تنسيقها بشكل أفضل:أنظمة معلومات محسّنة ذات معرّفات فريدة لتتبع ومراقبة الأفراد عبر الخدمات الصحية ومسار الحياة؛ وتعزيز استخدام البيانات دون الوطنية؛ وسلاسل إمداد أكثر مساءلة توفر مجموعة واسعة من الخدمات؛ وخدمات مجتمعية وقوى عاملة مدعمة.نحن نزعم أن الاستجابة لكل من فيروس نقص المناعة البشرية، والتهديدات الصحية الأوسع نطاقاً، ينبغي أن تعتمد على هذه المجالات للتقارب من أجل زيادة كفاءة النظم الصحية، وتخفيف ضرر أي انخفاض محتمل في التمويل الصحي.ستكون هناك حاجة إلى مزيد من الاستثمارات في تنفيذ ومراقبة عناصر هذه البرنامج، لإحراز تقدم نحو كل من التغطية الصحية الشاملة ومكافحة فيروس نقص المناعة البشرية.

## 摘要

基于地点、风险、临床特征和疾病负担的地理空间健康数据的改进以及专门的艾滋病 (HIV) 检测、预防和治疗，使得应对艾滋病病毒的工作有了更为微观的目标。这些方法显示对艾滋病疫情的控制有望实现。同时，联合国各成员国已承诺到 2030 年实现更广泛的健康和发展目标，其中包括全民健康覆盖 (UHC)。对艾滋病疫情有所控制将避免对艾滋病服务需求投入资源的不断增加，从而推进全民健康覆盖。然而，过度针对性的艾滋病应对措施也会干扰医疗系统，阻碍一体化并可能危及更广泛健康目标的实现。我们讨论了当前实现全民健康覆盖和艾滋病疫情控制的方法，指明了针对特定疾病的微观目标与整合的医疗系统间可能会有冲突的领域，并着重强调了促进这两项举措融合的机会。更好地协调这些有关计划要素的示例有：改进的信息系统，具有可在医疗服务和整个生命历程中跟踪并监控个人的唯一标识符；强化对地方数据的使用；用于提供广泛服务的更负责任的供应链；并加强基于社区的服务和员工培养。我们认为，对艾滋病和更广泛的健康威胁的应对措施都应使用此类融合领域，以提高医疗系统的效率并减轻医疗资金可能减少的危害。为了在全民健康覆盖和艾滋病疫情控制方面取得进展，需要在此类计划要素的执行和监测方面进一步投资。

## Резюме

Совершенствование сбора геопространственных данных о состоянии здоровья населения и индивидуализация процессов тестирования, лечения и профилактики ВИЧ привели к повышению микроадресации мероприятий по борьбе с ВИЧ с учетом местоположения, риска, клинического состояния и бремени заболевания. Эти подходы позволяют надеяться на обеспечение эффективного контроля над эпидемией ВИЧ. В то же время государства-члены ООН взяли на себя обязательства по расширению целей в области здравоохранения и развития к 2030 году, включая обеспечение всеобщего охвата услугами здравоохранения (UHC). Контроль над эпидемией ВИЧ будет содействовать обеспечению UHC, поскольку отпадет необходимость направлять постоянно растущее количество ресурсов на борьбу с ВИЧ. Однако слишком целенаправленный характер мероприятий по борьбе с ВИЧ может перекосить системы здравоохранения, замедлить процессы интеграции и несет в себе потенциальную угрозу достижению более широких целей в сфере здравоохранения. Авторы обсуждают существующие подходы к обеспечению UHC и контроля над эпидемией ВИЧ, отмечая потенциальные области конфликта между микроадресацией для конкретных заболеваний и объединенными системами здравоохранения, а также выявляя возможности для конвергенции, которые бы содействовали реализации обеих инициатив. Необходимо обеспечить согласованность следующих элементов программы: усовершенствованных информационных систем с уникальными идентификаторами для отслеживания и мониторинга отдельных лиц и оказываемых им медицинских услуг на протяжении всей жизни; более активного использования данных на субнациональном уровне; обеспечения жесткого учета и контроля в цепочках поставок для широкого спектра услуг, а также укрепления системы услуг, оказываемых по месту проживания, и развития соответствующих трудовых ресурсов. Авторы отмечают, что меры по борьбе с ВИЧ и другими угрозами в области здравоохранения должны опираться на эти области конвергенции с целью повышения эффективности систем здравоохранения и уменьшения вреда в результате потенциального сокращения финансирования. Необходимы дальнейшие инвестиции в реализацию и мониторинг этих программных элементов, чтобы добиться прогресса в обеспечении UHC и контроля над эпидемией ВИЧ.

## Introduction

As the global human immunodeficiency virus (HIV) response matures, national programmes in low- and middle-income countries are providing lifesaving treatment for more than 20 million people and reaching millions more each year with prevention interventions.^1^ This progress has been achieved with support from donors such as the United States President’s Emergency Plan for AIDS Relief and the Global Fund for AIDS, TB and Malaria. These investments have led to huge gains, with HIV-related mortality reduced by half compared with 2005 levels and a declining incidence of new infections in many countries and regions.^1^

The next phase of the HIV response is being driven by programmatic and technological innovations. Through improvements in data systems, geospatial mapping technologies and the use of large-scale population-based surveys, epidemiologists are now able to identify mismatches among burden of disease, size of populations most at risk and the availability of HIV testing, treatment and prevention services. These insights have led to programmes focusing more on subnational geographical units and on the HIV-related needs of specific populations, often referred to as microtargeting.^2^ Prevention services are increasingly tailored (or microtargeted) to specific locations and subpopulations based on their risks and through the identification of so-called hotspots where there are higher than usual rates of HIV incidence.^3^ Similarly, HIV treatment models are being differentiated based on patient characteristics and context to optimize quality and efficiency, while the allocation of HIV-specific funding and the intensity of HIV services have become more deliberately targeted.^4^

While HIV programmes have embraced this greater precision to maximize their impact, the national health systems of which they are a part have simultaneously committed to broader objectives. In ratifying the sustainable development goals (SDGs), United Nations Member States have pledged to achieve a series of ambitious health and development goals.^5^ In addition to ending acquired immune deficiency syndrome (AIDS) as a public health threat, SDG3 includes a 90% reduction in tuberculosis and malaria deaths, a one-third reduction in premature deaths due to noncommunicable diseases and achieving universal health coverage (UHC). UHC is the broadest of these goals, encompassing the other health-related SDGs, and is defined by the World Health Organization (WHO) as a condition in which “all people and communities can use the promotive, preventive, curative, rehabilitative and palliative health services they need, of sufficient quality to be effective, while also ensuring that the use of these services does not expose the user to financial hardship.”^6^ UHC includes equitable access to quality essential health-care services and to safe, effective and affordable essential medicines and vaccines, and financial risk protection.^6^

In many low- and middle-income countries, efforts to control the HIV epidemic and to achieve UHC are aligned and complementary; only by averting a growing population of citizens in need of HIV services can health systems hope to achieve universal coverage. The HIV response has also built capacity and programme infrastructure that can be used to address other health conditions. The advantages of integrating HIV, tuberculosis, primary care and other health services are becoming increasingly clear.^7^^,^^8^ Yet new trends in HIV programmes towards dynamic targeting of specific populations and regions, at a time of reductions in vertical funding, may or may not complement broader health goals such as UHC. This potential tension has not been widely explored. Our objective is to discuss these trends, identify potential areas of friction between HIV microtargeting strategies and the advancement of the UHC agenda, and highlight and recommend programme and policy actions to achieve greater convergence and health impact.

## Integration to achieve UHC

Major gains over the last two decades against disease-specific health threats have encouraged the global community to revisit the goal of health for all in the form of UHC, a foundational goal of the SDGs. According to the World Bank and WHO, the focus on UHC within the SDGs “provides a platform for an integrated approach within the health sector.”^6^ Central principles of the implementation of UHC are strengthened primary care,^9^ equity^6^ and promotion of service integration, defined by WHO as “the organization and management of health services so that people get the care they need, when they need it, in ways that are user-friendly, achieve the desired results and provide value for money.”^10^

Integration of clinical services for diverse health conditions, commonly at the primary health-care level, has generally been associated with positive outcomes for health care and process (e.g. patient satisfaction), without incurring additional costs.^11^ For instance, as HIV treatment simplified over the last decade (e.g. one pill, once daily), care became increasingly decentralized and delivered by non-physician clinicians,^12^ enabling integration with primary-care health services in some settings. Successful outcomes have been achieved via integration of HIV testing, prevention and treatment services with services for antenatal care, maternal and child health, sexual and reproductive health, tuberculosis and primary care at the point of care.^13^^–^^18^ Additionally, since HIV is the first chronic disease to be run as a successful national programme in many settings, there is growing interest in using the lessons and resources of its scale-up to strengthen noncommunicable disease programmes and to provide these services to patients enrolled in HIV programmes.^19^^,^^20^ The trend towards integration of HIV and other key services at the clinic level is well established, more responsive to an individual’s comprehensive needs (that is, more patient-centred) and strongly recommended by WHO.^21^ Yet more attention is required to determine the best approaches for managing the upstream systems on which provision of high-quality care relies.

The potential unintended risks of service-level integration include a loss of focus on individual disease responses (e.g. HIV, tuberculosis), which in theory could lead to underinvestment in disease-specific service delivery and programme monitoring.^22^ Nevertheless, service integration is likely a trend that will continue to accelerate to provide more sustainable people-centred services and the broader health benefits of UHC.

## Microtargeting responses

Advances in our understanding of HIV have led to the realization that we are dealing with not one, but hundreds of different epidemics. Even in what had previously been considered generalized epidemics, HIV is often distributed in localized clusters. For example, the United Republic of Tanzania’s HIV epidemic is driven by urbanization, transport routes, employment prospects and occupational locations (e.g. fishing), and subgroups of key populations.^23^ Furthermore, advances in HIV treatment access and greater programme maturity have led to an emphasis on more people-centred approaches that better meet individuals’ needs for HIV services, with the goal of increasing patients’ retention in programmes and thereby gaining efficiency. These trends have led to increased targeting of prevention and treatment programmes to maximize the public health impact with existing resources in the shortest possible time.

### Prevention interventions

Researchers and policy-makers are actively exploring the benefits and risks of targeting prevention interventions. A Kenyan study compared investment approaches based on uniform application of HIV prevention interventions versus a targeted approach.^4^ Projection models that integrate spatial analyses, transmission dynamic modelling of HIV and economic evaluation indicated that combination prevention strategies tailored to the risk behaviours of groups and their location could prevent substantially more infections for the same investment.^24^ This targeted approach, which was codified within the Kenya HIV Prevention Revolution Roadmap in 2014, was followed by a decline in HIV incidence.^25^^,^^26^

### Treatment interventions

Spatial and subnational data approaches are also being used to target HIV treatment towards areas of the highest disease burden.^27^ In Brazil, a unique identifier used across the public health system has enabled mapping of the spatial distribution of cumulative numbers of patients with HIV, the incidence of HIV, viral loads and key infected populations. These data showed that most of AIDS cases were in less than 10% of the country’s 5570 municipalities, which allowed for better targeting of resources.^28^

Beyond targeting based on geography, cost–effectiveness and risk groups, better data on patient needs and outcomes have led to differentiated service delivery strategies that further tailor (or micro-adapt) care to subgroups. Examples of such groups include patients considered clinically stable or unstable or those such as adolescents and key populations who benefit from customized service delivery approaches.^29^^,^^30^ Early results from programmes have indicated excellent retention results for clinically stable patients opting into less-intensive models of care delivery.^31^ These differentiated service delivery models may have a greater impact using existing resources if the projections of decreased costs (and greater effectiveness) are realized.^32^

### External resources

Major donors to the HIV response have adopted microtargeting approaches to their funding decisions. The United States President’s Emergency Plan for AIDS Relief’s strategy calls for United States Government resources to be applied to higher-burden geographical areas and health facilities (the right place), and also stresses the element of fast and efficient timing (the right time).^33^ Similarly, the Global Fund strategy emphasizes an operational focus on the highest burden countries and populations.^34^ These strategies focus on the best value for money for the HIV response from the donor perspective.

### Risks of microtargeting

There are potential unintended risks of microtargeting the HIV response. Heat maps that show concentrations of people on treatment or new HIV diagnoses may accurately highlight the need for additional HIV prevention and treatment services in high-burden areas. However, insufficient funding may mean that programmes are simply transferred away from areas of lower burden that still account for a substantial proportion of HIV infections. This issue was highlighted in the results of a mathematical model that supported targeting of prevention interventions overall, but noted that “75% of HIV seroconversions still occur outside the identified incidence clusters.”^35^ Focusing programmes based on the geographical concentration of disease may also mask the importance of epidemics within specific subgroups, and over-differentiating care models based on a large number of clinical characteristics could complicate delivery at scale. Furthermore, incomplete surveillance could lead to misleading assessments of the disease burden, which could threaten the degree to which greater equity of services can be achieved. The prerequisites of effective microtargeting therefore include the availability of accurate and complete data on HIV risks and programme outcomes at the subnational regions being considered, and the choice of relevant and unambiguous epidemiological and programme-based metrics or indicators to guide targeting.

There are clear benefits to targeted programmes, but policy-makers and programme managers need to ensure that these efforts are focused on greater equity and effectiveness, and do not undermine the strength of the public health approach, which is characterized by simple, streamlined, evidence-based strategies.^36^ Microtargeting strategies that include differentiated service delivery may therefore move away from this one-size-fits-all approach, for good reasons. However, unless scalable models can be developed, microtargeting may be difficult to implement widely in lower-resourced health systems, challenging to integrate with simpler primary health-care services and less sustainable from the perspective of domestic financing.

## Investing in convergence

Microtargeting for HIV care (whether by geography, population type or service delivery model) and the broader goals inherent in the UHC movement may appear to be in conflict. Yet it seems likely that both are necessary to achieve broader health goals. An improved understanding of potential differences and shared aims between these models can inform our strategies for achieving control of the HIV epidemic and the broader goal of UHC.

### Areas of potential divergence

Microtargeting of HIV services and the broader vision of health services that characterizes UHC could appear to diverge in their aims or implementation approaches. For instance, as shown in Table 1, coverage for integrated services is more likely to be driven by concerns for broad equitable access and parity of resources between regions and populations. HIV microtargeting on the other hand encourages differential coverage based on geography, HIV transmission and mortality risk, or severity of illness. Conversely, it could be argued that in some cases targeting may help to enhance the equity of the HIV response, particularly for individuals such as sexual minorities and others marginalized by existing health systems. However, greater equity through microtargeting would depend on local access to data on risks or needs among these subpopulations and prioritization of its use.

**Table 1:** Areas of potential divergence between human immunodeficiency virus programme microtargeting and broader goals of universal health coverage

HIV programme microtargeting	Domain	Integrative strategies for SDGs and UHC
Geographically and risk-focused coverage of specific interventions (e.g. pre-exposure prophylaxis programmes for urban sex workers)	Programme coverage	Broad-based equal access to integrated prevention and treatment services for common illnesses and conditions
Dynamic and potentially frequent shifts in interventions and funding driven by data suggesting changes in geographic and population concentrations of the epidemic and response	Consistency of programming	Regular access to services for all populations and conditions (e.g. for antenatal care, diagnosis and treatment of hypertension, treatment for childhood diarrhoeal disease)
Stigma and discrimination around acknowledging and engaging key populations (e.g. sex workers, individuals who inject drugs)	Level of stigma and discrimination	Services are less targeted and less affected by stigma and discrimination
Strong donor imperative to reach targets and show success	Degree of investment and influence	Generally financed by domestic or out-of-pocket funding with less external accountability
Time pressure to meet coverage targets to achieve well defined goals for controlling the HIV epidemic	Definition and urgency of meeting goals	The urgency around achieving of UHC generally remains less well defined and understood than disease-specific programmes

HIV: human immunodeficiency virus; SDG: sustainable development goals; UHC: universal health coverage.

Successful microtargeting will require a dynamic environment with rapid shifts in strategies and resource allocation, analogous to an outbreak response. For example, scaling-up the use of assays that enable identification of recent HIV infections will make it possible to identify and shift HIV programme support to communities or groups experiencing outbreaks of new infections.^37^ In contrast, systems that deliver primary care for routine acute and chronic diseases (the core of UHC) require consistent support, but generally have far fewer resources for implementation. Provision of basic services may depend on the additional staff, newer data systems or increased attention to supply chains provided by a vertical programme. This reliance leaves those core services vulnerable if a disease-specific programme responds to new data by swiftly pivoting away from a geographical area. Funding for UHC is typically more reliant on domestic government expenditure, national health insurance schemes or out-of-pocket costs, whereas a larger proportion of the HIV response remains externally financed. This arrangement leads to greater external accountability of the HIV programme response, but can also threaten the ability to shift the programmes to local ownership if programmes are not built in a way that can be sustained within the local health system.^38^

### Achieving greater health impact

Closer examination of HIV microtargeting and the movement towards greater integration of services for UHC suggests areas of convergence that could help to mitigate the effects of differing approaches. At the core of this convergence is the basic idea that HIV programmes occur within health systems and must align with national health goals; HIV epidemic control cannot come at the expense of broader health outcomes. The converse is also true; in many countries, desired reductions in population morbidity and mortality cannot be achieved in the absence of HIV epidemic control. Effective HIV microtargeting should lead to faster attainment of HIV-specific goals and less medium- or long-term need for HIV testing, prevention and treatment services. In the long term, at least, these impacts will free-up health systems to support broader UHC goals.

More immediately, the health-system building blocks needed to deliver both HIV microtargeting strategies and broader UHC services have features in common, including quantity, quality and distribution of health-care workers as well as laboratory, supply chain and information systems (Box 1). While the systems built for one response will not automatically provide benefits more broadly, strategic and intentional investments that promote shared benefits may make this possible. For example, investments in upgraded national information systems to include unique patient identifiers and the ability to track individuals longitudinally are essential for both targeted HIV strategies and for UHC. Systems built initially for a data-driven, targeted HIV response can be used to support services for other diseases and conditions of public health concern. These systems could include tracking changes in demand for sexual and reproductive health services and family planning coverage for high-risk subgroups or responding to an acute disease outbreak like Ebola virus disease.

Box 1 **Areas of potential convergence between human immunodeficiency virus programme microtargeting and broader goals of universal health coverage**

**Broader beneficial effects of HIV control**

Efficient reductions in new HIV infections will result in less need for lifelong HIV treatment services, thereby reducing the burden on health systems and freeing up resources for other health priorities.

**Use of common clinical platforms**

Stronger primary health-care systems, if prioritized through national UHC financing strategies, provide additional routes to deliver targeted HIV services to those patients with less intense clinical needs.

**Health-care worker performance**

Improvements in national systems would support pre-service education and performance management (e.g. systems of incentivizing, mentoring, supervising) for health-care workers.

**Information systems and data use**

Responsive electronic information systems (e.g. systems that are networked, include unique patient identifiers and promote subnational data use) are fundamental to both targeted HIV interventions and outcome-based programming for noncommunicable diseases, civil registration and vital statistics programmes and other elements of UHC.

**Laboratory systems**

Improvements in laboratory systems (e.g. equipment, sample transportation systems, staff and information systems) through microtargeting of high-volume sites for HIV service delivery could benefit UHC delivery and management of other noncommunicable diseases throughout a region.

**Community delivery systems and civil society**

Microtargeting of HIV services as well as integrated disease management and prevention services are highly reliant on well managed community systems to deliver focused messages and interventions into communities, with support from civil society.

**Supply-chain management**

HIV microtargeting and many UHC goals require strong, yet responsive, supply chains that are held accountable by providers and society. Greater integration of health services and joint performance management could yield substantial health benefits.

Human immunodeficiency virus (HIV); universal health coverage (UHC).

Major donors have recognized the importance of this strategy and have increased investments in many of the areas that can be considered convergent. For instance, the Global Fund has sponsored several rounds of funding specifically aimed at improving health systems’ resilience, and its 2017–2022 strategy calls for further such investments.^34^ Similarly, nearly all the grants of the United States President’s Emergency Plan for AIDS Relief include cross-cutting health systems investments, which are targeted increasingly towards areas of weakness in the Plan’s sustainability index and dashboard.^39^ This tool includes 90 domains and ranks areas, including commodity security, supply chains and laboratory services. Donors for broader UHC goals, such as Gavi, the Vaccine Alliance and the Global Financing Facility, also make investments in strengthening health systems. However, the currently limited coordination and use of health-systems investments across disease-specific responses could be improved.

In addition to broader financing initiatives and governance strategies, as explored by others,^7^ we believe that more systematic measurement of the functional performance of health system elements is essential for greater impact. The recently launched global Primary Health Care Performance Initiative,^40^ and the related development of primary care vital-signs indicators that are oriented towards systems and outcomes (e.g. a service quality index), may be a step in the right direction, especially if they are collected subnationally and disaggregated by population types. While HIV programme managers may not see the connection between their work and a primary health-care indicator like vaccination coverage, they may see the benefit of leveraging one another’s programming to strengthen the overall supply chain. Similarly, global actors such as the World Bank and national governments are using the Vital Statistics Performance Index to monitor national progress in developing the civil registration and vital statistics systems that are fundamental to both disease-specific and broader UHC goals.^41^^,^^42^ Such systems monitoring should, in theory, sharpen the tracking and accountability for the effectiveness of investments in these areas, and encourage further investment and policy change.

Similar approaches to measuring performance could be more systematically applied to other areas of convergency shown in Box 1. For example, community-based service delivery systems are important for microtargeting strategies for HIV prevention and treatment as well as UHC and integration goals.^21^^,^^43^ Nevertheless, recent studies have found workforces to be poorly coordinated and integrated across disease-specific responses, and inadequately harmonized with national goals.^44^^,^^45^ Recognizing this challenge, WHO intends to develop guidelines on health policy and systems support for community health worker programmes. However, there is no recognized approach to monitoring the performance of community-based care delivery systems across the full range of health responses for which they are deployed. Similarly, there are no routinely used tools for system-wide performance monitoring of national information systems or supply chains for multiple diseases or conditions and similar systems, within or among countries. These are high priority areas for future research. The value of developing and validating high-quality performance measures would be greater accountability for the effectiveness of health systems investments, which could ultimately reduce the need for parallel disease-specific systems and result in greater impact. A series of commonly accepted indices could also provide a greater incentive for greater cross-donor and disease co-investment in these basic elements of sustainable health responses, even if resources decrease.

## Conclusions

Improvements in geospatial data and HIV testing, prevention and treatment services have led to microtargeting within the HIV response, based on location, population risk and illness severity. Although these approaches show great promise for achieving control of the HIV epidemic, which is fundamental to the achievement of the SDGs, there are potential risks to broader health systems goals unless specific actions are taken. To maximize synergies among programmes, leaders of the HIV and UHC responses should recognize opportunities for programming in areas of convergence. Committing to using each other’s programmes and resources would have a greater collective impact on health. Further investment and enhanced approaches to performance measurement of these convergent elements will be critical to achieve both sustainable control of the HIV epidemic and the broader goals of UHC.
